# The multi-faceted role of NADPH in regulating inflammation in activated myeloid cells

**DOI:** 10.3389/fimmu.2023.1328484

**Published:** 2023-12-01

**Authors:** Kenneth K. Y. Ting, Jenny Jongstra-Bilen, Myron I. Cybulsky

**Affiliations:** ^1^Department of Immunology, University of Toronto, Toronto, ON, Canada; ^2^Toronto General Hospital Research Institute, University Health Network, Toronto, ON, Canada; ^3^Department of Laboratory Medicine and Pathobiology, University of Toronto, Toronto, ON, Canada; ^4^Peter Munk Cardiac Centre, University Health Network, Toronto, ON, Canada

**Keywords:** NADPH, macrophages, LPS (lipopolysaccharide), myeloid cells, immunometabolism, dendritic cells, cofactors, cofactor coordination

## Abstract

Recent advances in the immunometabolism field have demonstrated the importance of metabolites in fine-tuning the inflammatory responses in myeloid cells. Cofactors, which are metabolites comprised of inorganic ions and organic molecules, may tightly or loosely bind to distinct sites of enzymes to catalyze a specific reaction. Since many enzymes that mediate inflammatory and anti-inflammatory processes require the same cofactors to function, this raises the possibility that under conditions where the abundance of these cofactors is limited, inflammatory and anti-inflammatory enzymes must compete with each other for the consumption of cofactors. Thus, this competition may reflect a naturally evolved mechanism to efficiently co-regulate inflammatory versus anti-inflammatory pathways, fine-tuning the extent of an inflammatory response. The role of NADPH, the reduced form of nicotinamide adenine dinucleotide phosphate (NADP^+^), in mediating inflammatory and anti-inflammatory responses in activated myeloid cells has been well-established in the past decades. However, how the dynamic of NADPH consumption mediates the co-regulation between individual inflammatory and anti-inflammatory pathways is only beginning to be appreciated. In this review, we will summarize the established roles of NADPH in supporting inflammatory and anti-inflammatory pathways, as well as highlight how the competition for NADPH consumption by these opposing pathways fine-tunes the inflammatory response in activated myeloid cells.

## 1 Introduction

Major milestones in the immunometabolism field have been recently reached to illustrate how intracellular metabolic circuits are rewired to orchestrate a fine-tuned inflammatory response in activated immune cells. For instance, in M1 pro-inflammatory macrophages (Mφs), there is a metabolic break at isocitrate dehydrogenase in the TCA cycle due to suppression of its mRNA expression, leading to the accumulation of citrate and itaconate, which drive lipid synthesis and stabilization of anti-inflammatory transcription factors, such as nuclear factor-erythroid factor 2-related factor 2 (NRF2) and activating transcription factor 3 (1–5). The accumulation of itaconate causes a metabolic break by inhibiting the enzymic activity of succinate dehydrogenase. The consequent increased abundance of succinate leads to the stabilization HIF-1α, which promotes IL-1β transcription (1–3, 6, 7). On the other hand, M2 anti-inflammatory Mφs primarily use fatty acid oxidation and oxidative phosphorylation to support their metabolism, although the upregulation of glycolysis by mTOR complex 2 (mTORC2), IL-4Rα/Stat6 and interferon regulatory factor 4 (IRF4) was also reported to be critical (8). Apart from this, carbohydrate kinase-like protein (CARKL) was also activated in M2 Mφs, which led to the enhancement of the non-oxidative branch of the pentose phosphate pathway (PPP) (9). This subsequently increased the synthesis of uridine diphosphate N-acetylglucosamine (UDP-GlcNAC), which is required for the N-glycosylation of many cell surface proteins expressed on M2 Mφs (2). Taken together, these studies revealed that metabolites can regulate both inflammatory and anti-inflammatory processes, in addition to intracellular metabolism and energetics. Although the moonlighting functions of these metabolites, including their roles in signaling, post-translational modification, and epigenetics are being increasingly appreciated (10), how they regulate the extent of inflammation by coregulating inflammatory and anti-inflammatory pathways remain unclear.

**Figure 1:**
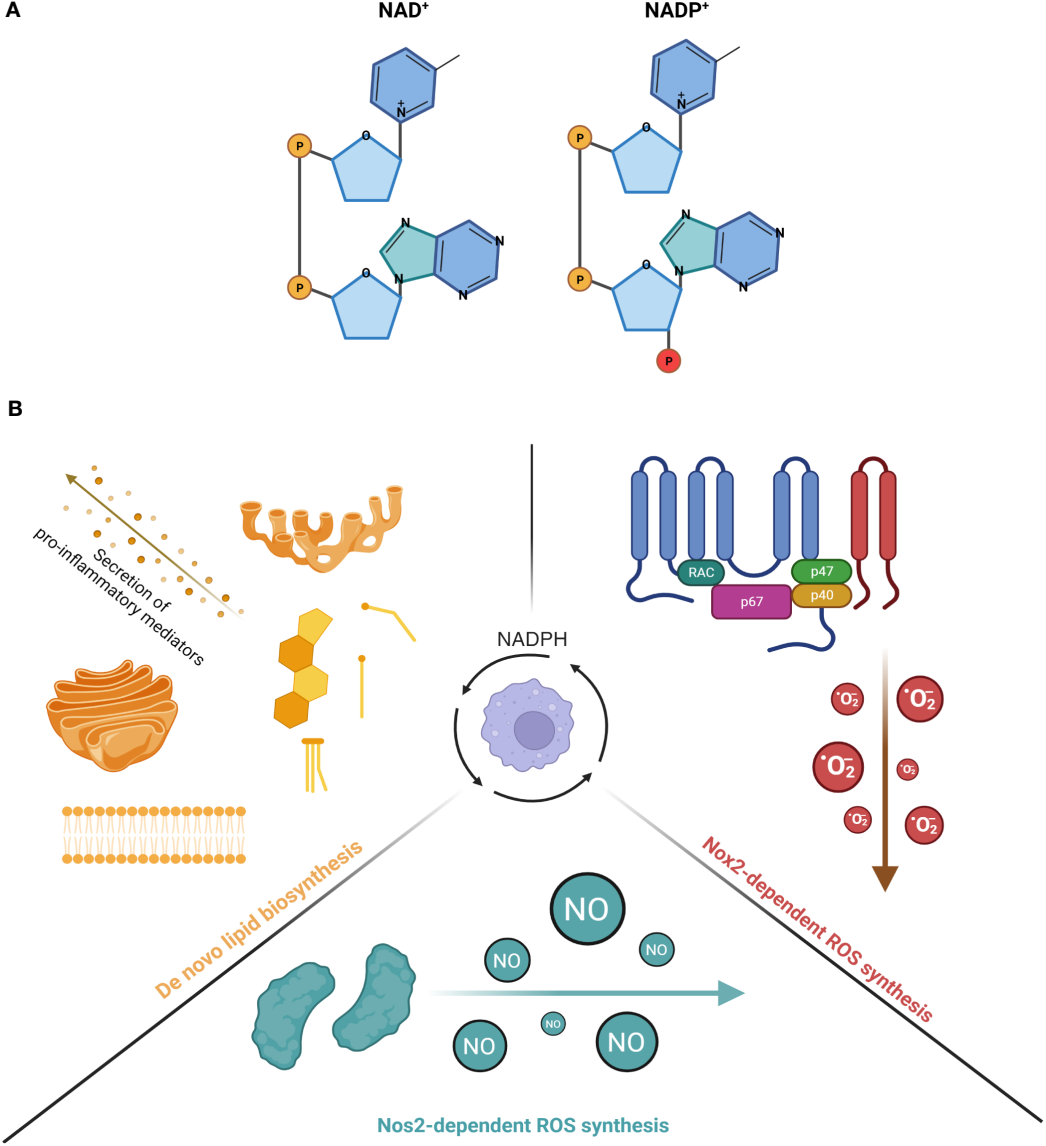
**(A)** The molecular structure of NAD^+^ and NADP^+^. Left diagram shows the structure of NAD^+^ while right diagram shows the structure of NADP^+^. The phosphate in red illustrates the additional phosphate on the 2' position of the ribose ring attached on NADP^+^. **(B)** The consumption of NADPH in activated myeloid cells for inflammatory processes. (Left) NADPH serves as a co-factor for many enzymes involved in the production of de novo lipid synthesis (steroids, cholesterol and fatty acids). The increased production of these lipid species is important for the membrane expansion of Golgi network and endoplasmic reticulum, a requirement for the secretion of pro-inflammatory cytokines. (Right) NADPH is an important co-factor for NOX2 and its generation of superoxide anions. NOX2 is a 6-subunit complex assembled on the plasma membrane that transfers one electron from NADPH, which is the primary substrate of the reaction, to oxygen, thereby forming O_2_^-^. Under basal conditions, the components of the complex are localized in different subcellular compartments, with the gp91phox and p22phox subunits localized on the plasma membrane as one heterodimeric complex, known as flavocytochrome b558 (cyt b558). On the other hand, the p47phox/p67phox/p40phox subunits are co-localized in the cytosol, forming another complex. Upon stimulation, p47phox is phosphorylated, which leads the cytosolic complex, together with small GTPase Rac1/Rac2, to bind to the flavocytochrome complex, ultimately forming the final oxidase. (Bottom) NADPH is a critical co-factor for NOS2 and its generation of nitric oxide (NO) in a two-step reaction. The first reaction involves 1 molecule of L-arginine as a substrate being oxidized at a guanidino nitrogen to produce Nω-OH-L-arginine as an intermediate. The second reaction involves Nω-OH-L-arginine being further oxidized to produce 1 molecule of NO and L-citrul-line. During both reactions, a total of 1.5 molecules of NADPH and 2 molecules of dioxygen, which are co-substrates of the reactions, are converted to 1.5 molecules of NADP+ and 2 molecules of water as co-products. Created with BioRender.com.

Unlike nicotinamide adenine dinucleotide (NAD^+^), nicotinamide adenine dinucleotide phosphate (NADP^+^) has an additional phosphate on the 2’ position of the ribose ring that attaches to an adenine moiety and has a lower intracellular concentration than NAD^+^ (11) (**Figure 1A**). The reduced form of NADP^+^, known as NADPH, is a well-established, indispensable cofactor required for anabolic reactions, oxidative and antioxidative processes. Although NADPH has multifunctional roles in regulating inflammation, particularly in myeloid cells that have been clearly defined in the past, it remains unclear how NADPH-dependent inflammatory and anti-inflammatory pathways are co-regulated in order to fine-tune the magnitude of an inflammatory response. In this review, we will revisit the traditional roles of NADPH in inflammatory and anti-inflammatory pathways and highlight studies that reveal the competition for its consumption between these opposing pathways as a way to regulate inflammation in myeloid cells.

## 2 NADPH usage for inflammatory processes

In general, NADPH supports a wide range of inflammatory processes in myeloid cells, including *de novo* lipid biosynthesis and generation of ROS (**Figure 1B**). For instance, Everts et al. demonstrated that in dendritic cells (DCs), LPS-induced glycolysis is repurposed to replenish citrate, an intermediate metabolite in the TCA cycle that is depleted for the *de novo* synthesis of fatty acids (12). This subsequently increased the synthesis of additional membranes to expand the endoplasmic reticulum (ER) and golgi networks, which is required for the secretion of proinflammatory lipid mediators, such as prostaglandin E_2_ (12). Similar to DCs, LPS-induced activation of Mφs also led to an upregulation of fatty acid synthesis (13, 14), which has now been shown to regulate the inflammatory responses of Mφs. For example, Carroll et al. demonstrated that the production of acetoacetyl-CoA by fatty acid synthase (FASN), a key NADPH-dependent enzyme involved in fatty acid synthesis, can regulate TLR signaling as acetoacetyl-CoA is linked to cholesterol synthesis and subsequently involved in modulating the formation of lipid rafts (15). Similar findings were also reported by Wei et al., who found that mice deficient of FASN are protected from diet-induced insulin resistance and inflammation as it altered the composition of plasma membrane and subsequently disrupted Rho GTPase trafficking, a process that is required for the activation of Mφs (16). Apart from lipid raft formation, reports also suggest that LPS-induced *de novo* lipogenesis is important for phagocytosis as it requires ongoing lipid synthesis in the ER for membrane expansion, such that Mφs can surround and capture targeted pathogens for internalization (17, 18). The mechanism that links lipogenesis with phagocytosis was later elucidated by Lee et al., who showed that phagocytosis was impaired in LPS-activated Mφs isolated from mice deficient in sterol regulatory element binding protein 1a (SREBP-1a) (19), which regulates the transcription of genes related to lipogenesis (20). Specifically, the study revealed that SREBP-1a-dependent lipid species mediate the interaction between membrane lipid rafts and the actin cytoskeleton, an association that is critical for the early stages of phagocytosis (19). Finally, SREBP-1a is also known to regulate genes that are related to NADPH synthesis (21), and Mφs with genetic deficiency of SREBP-1a demonstrated decreased cytokine production and inflammasome activation upon challenge by pro-inflammatory stimuli (22). Collectively, these studies have demonstrated the importance of *de novo* lipogenesis, an anabolic process that is NADPH-dependent, in regulating the inflammatory response of myeloid cells.

Apart from lipid biosynthesis, another critical pro-inflammatory process that NADPH supports in myeloid cells is the production of cytotoxic, diffusive reactive radicals, such as reactive oxygen species (ROS) via NADPH oxidases (NOXes), as well as nitric oxide (^·^NO) via nitric oxide synthases (NOSes). Indeed, mice with defects of NOX2 or NOS2 failed to restrain bacterial replication (23, 24). The importance of ROS generation by NOX2, including superoxide anions (O_2_^-^) and hydrogen peroxide (H_2_O_2_), has been extensively reviewed in the past (25, 26). Apart from Mφs, a new study now revealed that PMA-activated neutrophils repurpose glycolysis and the PPP in order to maximize the yield of NADPH from glucose metabolism (27). This adaptation is required to meet the high demands of NAPDH needed for the oxidative burst mediated by NOXes (27). Taken together, these studies have shown the significance of NADPH-dependent production of ROS via NOXes in regulating the inflammatory response in myeloid cells. Upon activation by pathogenic microorganisms, Mφs produce a burst of ROS and NO that limit bacterial infection in the host (23, 28, 29). Low levels of ROS act as second messengers for activating inflammatory intracellular signaling, such as NF-κB and MAP kinase pathways (30).

Similar to the production of ROS by NOXes, the production of NO by NOSes is also dependent on NADPH. Specifically, three genes encode NOSes in mammals: *Nos1, Nos2* and *Nos3*. NOS2 is also known as iNOS (“i” refers to its immunologically inducible nature) and was first cloned in Mφs (31). It is only expressed in cells that are activated by proinflammatory cytokines or stimuli. NOS2 function is not regulated by the elevation of intracellular Ca^2+^. The production of NO by NOS2 (in micromolar amounts) is much higher and more sustained than by other NOSes, thereby making NOS2 an important player in regulating inflammation and infection (28, 32). Regardless of the NOS isoform, the biochemical pathway to produce NO is the same and all require cofactors including NADPH, flavin adenine dinucleotide (FAD), flavin mononucleotide (FMN), tetrahydrobiopterin (BH_4_) and ferrous iron (Fe^2+^). NADPH is of primary importance as its selective omission mostly impaired the activity of NOS2 in activated Mφs (33).

## 3 NADPH usage for antioxidative purposes

**Figure 2:**
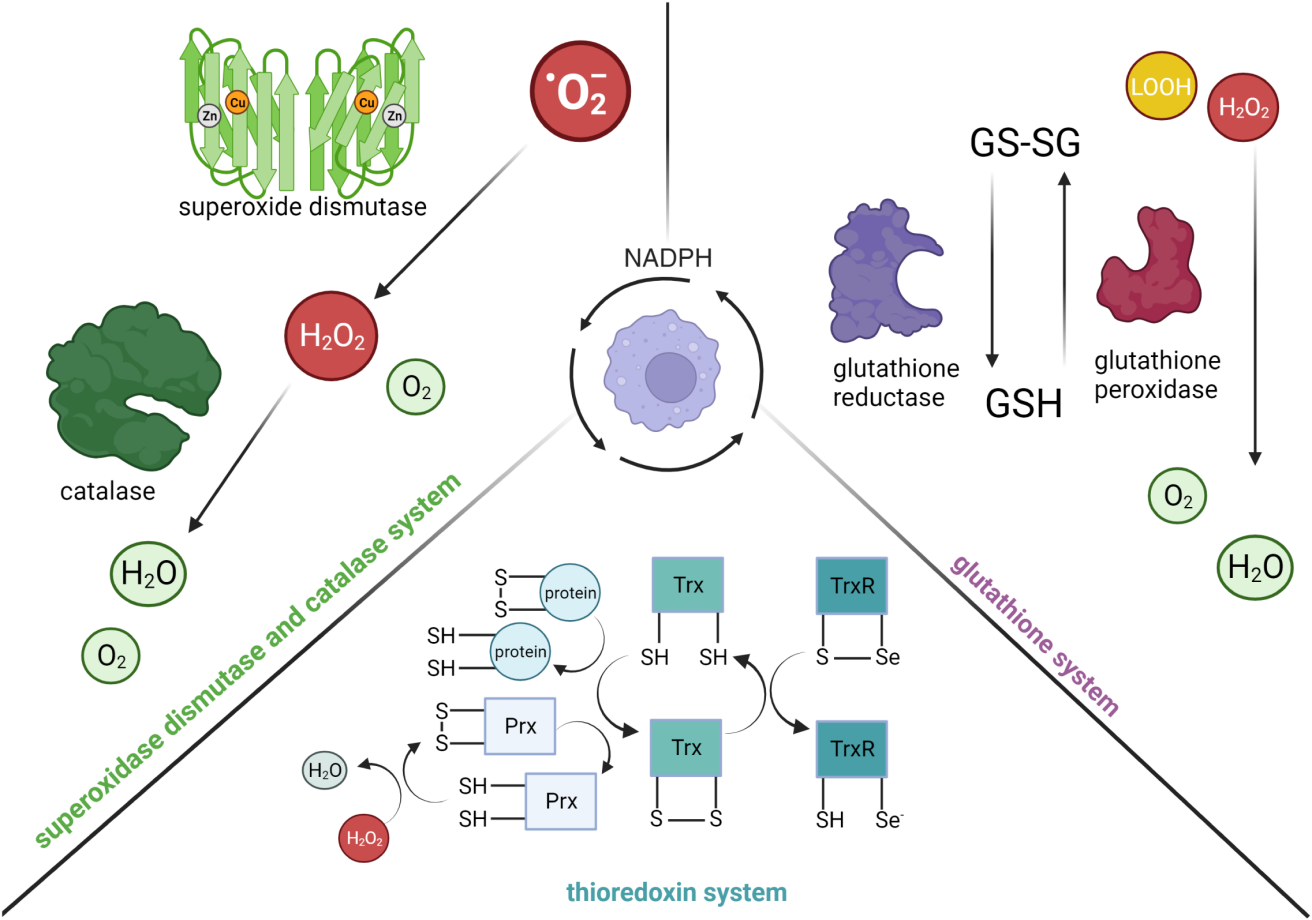
The consumption of NADPH in activated myeloid cells for anti-inflammatory processes. (Left) Superoxide dismutase detoxifies superoxide anions (^·^O_2_^-^) into hydrogen peroxide (H_2_O_2_) and oxygen (O_2_). Binding of NADPH to catalase is critical for activating its enzymatic function, specifically the conversion of hydrogen peroxide to water. (Right) Glutathione reductase reduces glutathione disulfide (GS-SG) to glutathione (GSH) in a NADPH-dependent manner. GSH is an important intracellular antioxidant used by glutathione peroxidase to reduce hydrogen peroxide or lipid hydroperoxide (LOOH) to water and oxygen. (Bottom) Thioredoxin reductase (TrxR) supports the reduction of thioredoxin (Trx(SH)_2_) proteins and peroxidases (Prx(SH)_2_) in a NADPH-dependent manner (selenium, Se). The reduction of peroxidases is important for the conversion of hydrogen peroxide to water, as well as reduction of oxidized proteins. Created with BioRender.com.

While cytotoxic reactive radicals produced by myeloid cells are essential for limiting bacterial replication, they can also be harmful to the host (34, 35). It is intriguing that NADPH-dependent detoxification pathways have evolved as defense mechanisms utilized by myeloid cells (**Figure 2**). In general, three major systems help to protect host cells from oxidative and nitrosative stress: (1) the superoxidase dismutase (SOD) and catalase system, (2) the glutathione system and (3) the thioredoxin system. Briefly, SOD converts superoxide anions to hydrogen peroxide and oxygen, which is then detoxified to water by catalase. After the discovery of SOD as the first line of defense against ROS (36), three SODs have been identified: SOD1 (cytoplasmic and peroxisome), SOD2 (mitochondrial) and SOD3 (extracellular matrix). Like SOD, three types of catalases have also been characterized (37), with the monofunctional heme-containing type being the most common (38). In a two-step reaction, catalase breaks down two hydrogen peroxide molecules, which are derived from the reaction catalyzed by SOD, into one molecule of oxygen and two molecules of water. While the heme group is critical for its activity, past studies have also demonstrated the requirement of a tightly bound NADPH to the active conformation (39, 40). Furthermore, given the important role of catalases in regulating ROS levels, its localization in the peroxisome (41, 42) has been linked to the modulation of innate immune signaling. For instance, the reduction of catalase in peroxisomes from Drosophila-derived Mφs was found to impair actin organization and phagocytic activity in a p38-MAPK-dependent manner (43).

Apart from the SOD and catalase system, the glutathione system, which is composed of glutathione (GSH), γ-glutamyl cysteine synthase (GCS), GSH synthetase, glutathione peroxidase, and glutathione reductase (GSR), also plays an important role in mediating cellular redox homeostasis. GSH is a tripeptide antioxidant, in which its synthesis is catalyzed by GCS and GSH synthetase from glutamate, cysteine and glycine. Cellular GSH is found mostly in the cytosol, with the remainder spread across organelles (44). Due to the cysteine residues of GSH, it can be readily oxidized to glutathione disulfide (GSSG) by electrophilic species directly (e.g., combine with NO to form less reactive S-nitrosoglutathione), or indirectly through enzymatic reactions (e.g., reduce hydrogen peroxide to water by glutathione peroxidase). GSSG can be reduced back to GSH by GSR in a NADPH-dependent manner.

The thioredoxin system is composed of Trx proteins and Trx reductases (TrxR), in which the reduced form of Trx proteins (Trx(SH)_2_) are disulfide reductases as they contain dithiol groups in their highly conserved active site (-Cys-Gly-Pro-Cys-) (45, 46). Specifically, the dithiol groups of Trx(SH)_2_ can directly reduce oxidized proteins or provide electrons to thiol-dependent peroxidases (Prx) to convert hydrogen peroxide to water. The oxidation of the dithiol groups of Trx(SH)_2_ consequently results in the formation of intra-chain disulfide bridges and hence the oxidized form of Trx proteins (TrxS_2_). The intra-chain disulfide bridges in TrxS_2_ can be reversibly reduced back to Trx(SH)_2_ by TrxR in a NADPH-dependent manner. To date, three isoforms of Trx have been identified in mammalian cells: Trx1 (cytosolic), Trx2 (mitochondrial), and SpTrx (spermatozoa cells) (47). Similar to Trx proteins, TrxR are also oxidoreductases that can catalyze reduction on small-molecule substrates, such as H_2_O_2_ and lipid hydroperoxide, in addition to TrxS_2_. Specifically, TrxR are selenocysteine-containing enzymes that utilize the reducing equivalents from NADPH to catalyze reduction reactions.

## 4 Competition for NADPH between proinflammatory and antioxidative processes

NADPH plays a dual role in regulating both oxidative and antioxidative processes during inflammation; thus, the abundance of NADPH is significantly limited during the activation of myeloid cells. For instance, Everts et al., showed a significant depletion of NADPH pools in LPS-activated DCs, while others also reported similar findings in Mφs stimulated with LPS alone, or LPS with IFN-γ (27, 33, 48). To regenerate more reduced equivalents of NADPH, LPS-activated Mφs upregulate NADPH-generating pathways, including glucose-6-phosphate dehydrogenase (G6PD), which is the rate limiting enzyme of the PPP (2, 49, 50). Specifically, it decarboxylates G6P and forms ribose-5-phosphate (R5P) via three irreversible reactions. During these reactions, two molecules of NADP^+^ are reduced to NADPH with the simultaneous liberation of one CO_2_ molecule. Like enzymes in glycolysis, G6PD is allosterically regulated. Under resting condition where there is a high NADPH/NADP^+^ ratio, G6PD remains as an inactive monomer since NADPH binds to its allosteric site. However, during inflammatory condition where there is a high demand to consume NADPH, G6PD is released from its inhibition and forms an active homodimer (51, 52). Apart from the PPP, two subtypes of isocitrate dehydrogenase (IDH) isoenzymes can also generate NADPH based on their intracellular localization: mitochondrial NADP^+^-dependent IDH (*Idh2*), as well as cytosolic and peroxisomal NADP^+^-dependent ICDH (*Idh1*). Both families of enzymes use NADP^+^ as cofactors to perform reversible reactions, where isocitrate is oxidatively decarboxylated to alpha-ketoglutarate and generates one NADPH per reaction. In the context of LPS-activated Mφs, the expression of *Idh1* has been conflicting as various studies have reported its expression to be increased (53), decreased (2) or unaffected (50). Finally, folate-mediated one carbon metabolism, in which its activity is induced in LPS-activated Mφs (54) also contributes to the regeneration of reduced NADPH levels, with serine and glycine as the major carbon sources of this pathway. Specifically, methylene tetrahydrofolate (THF) dehydrogenases catalyze the oxidation of 5,10-methylene-THF to form 10-formyl-THF, which is subsequently oxidized to CO_2_ with concomitant NADPH production by 10-formyl-THF dehydrogenases (55). Overall, all the studies above have shown that upon inflammatory activation of myeloid cells, there is a high demand for NADPH consumption. This leads to its marked depletion with a concomitant increase of activity in the PPP as the primary mechanism to generate more reducing equivalents of NADPH.

Since the concentration of NADPH in resting Mφs is in the micromolar range (33), which is within the range of the K_m_’s of NADPH-dependent enzymes, such as NOS2 (33), NOXes (56), glutathione reductase (57), small changes in intracellular NADPH abundance will greatly impact the activity of these enzymes (58). Therefore, under inflammatory conditions where NADPH is even further depleted, these enzymes may compete for reduced NADPH equivalents. Indeed, one study has shown that administrating Kuppfer cells with *t*-butyl hydroperoxide, a substrate for glutathione peroxidase, inhibited the production of superoxide (59), which implied that the increased activity of the antioxidative pathway limits the availability of NADPH for the use by oxidative pathways (58). In addition, recently we showed that NADPH consumption by HIF-1α versus NRF2-dependent apoenzymes is vital for regulating inflammation in Mφs (60). Specifically, the accumulation of oxidized low-density lipoprotein (oxLDL) in Mφs enhanced LPS-induced expression of NRF2-dependent ROS detoxification enzymes (i.e., GSR) and suppressed the expression of HIF-1α-dependent ROS producing enzymes (i.e., NOS2). This subsequently led to a shift of NADPH consumption from oxidative to antioxidative processes, eventually impairing the inflammatory responses in Mφs with accumulated oxLDL.

Apart from this, the competition between NADPH-dependent enzymes in inflammatory versus antioxidative pathways can also be revealed by blocking the function of G6PD under inflammatory conditions as the output of both pathways will be impaired. For instance, for inflammatory pathways, blocking the expression of G6PD impaired pro-inflammatory cytokine expression and lipid accumulation in LPS-activated DCs (12). In addition to *de novo* lipid synthesis, the loss of G6PD function also led to impaired NOS2 activity in Mφs activated by LPS (61) or by IFN-γ and infected by *Trypanosoma cruzi* (62). Human granulocytes that are deficient of G6PD also have impaired production of superoxide, nitric oxide, and hydrogen peroxide (63). Similar findings were also reported in PMA-stimulated mouse and human neutrophils where inhibition of G6PD significantly impaired their ability to undergo oxidative burst (64).

## 5 Future perspectives and conclusion

Many studies in the last few decades have identified and characterized the multifunctional roles that NADPH plays in regulating inflammation, redox homeostasis, and anabolic processes. However, how NADPH simultaneously coordinates these disparate functions to regulate the extent of an inflammatory response remains unclear. In this review, we have highlighted studies that demonstrate how NADPH metabolism is altered in activated myeloid cells, and how the competition for NADPH consumption between oxidative and antioxidative pathways reflects a potential way to efficiently regulate the magnitude of inflammation. More importantly, recent technological advancements have enabled the development of tools to quantify and trace NADPH levels in real-time and across subcellular compartments, thereby providing spatial and temporal information that was previously unavailable with traditional methodologies (65–68). For instance, quantitative flux analysis of NADPH, which employs tracking of deuterium incorporation into NADPH, has revealed that folate-dependent methylenetetrahydrofolate dehydrogenase (MTHFD)-mediated NADPH production provides anti-oxidant activity to cells and enables resistance to oxidative stress (68). Not surprisingly, many human cancer cells overexpress genes of the *MTHFD* family (69) and thus are promising targets for anti-cancer therapeutics.

To date, NADPH metabolism has been targeted primarily for cancer therapeutics as cancer cells upregulate NADPH synthesis to support their massive antioxidative and anabolic requirements (55). The differential metabolic requirements between cancerous and non-cancerous cells provides a therapeutic opportunity for regulating selective cellular immune responses. Indeed, inhibitors that target NADPH synthesis enzymes, which aim to manipulate ROS levels and induce cell death selectively in cancerous cells, have been extensively developed (55). Several inhibitors of G6PD and IDH, such as RRx-001, DHEA and AG-881, have demonstrated promising efficacy and have entered Phase III clinical trials (55). However, the synthesis of highly selective or isoform-specific inhibitors that reduce unwanted side effects still remains challenging. Future research is warranted to address these challenges and investigate the possibility of synergizing inhibitors of NADPH synthesis for novel combinatorial therapies with current chemotherapeutics.

## Author contributions

KKYT: Writing – original draft, Writing – review & editing. JJ-B: Writing – review & editing. MIC: Writing – review & editing.
